# ω-3 PUFAs ameliorate liver fibrosis and inhibit hepatic stellate cells proliferation and activation by promoting YAP/TAZ degradation

**DOI:** 10.1038/srep30029

**Published:** 2016-07-20

**Authors:** Kun Zhang, Yanan Chang, Zhemin Shi, Xiaohui Han, Yawei Han, Qingbin Yao, Zhimei Hu, Hongmei Cui, Lina Zheng, Tao Han, Wei Hong

**Affiliations:** 1 Department of Histology and Embryology, School of Basic Medical Sciences, Tianjin Medical University, Tianjin 300070, China; 2 The Third Central Clinical College of Tianjin Medical University, Tianjin 300170, China; 3 Department of Hepatology, Tianjin Third Central Hospital, Tianjin 300170, China; 4 Tianjin Key Laboratory of Artificial Cells, Tianjin 300170, China

## Abstract

Elevated levels of the transcriptional regulators Yes-associated protein (YAP) and transcriptional coactivators with PDZ-binding motif (TAZ), key effectors of the Hippo pathway, have been shown to play essential roles in controlling liver cell fate and the activation of hepatic stellate cells (HSCs). The dietary intake of omega-3 polyunsaturated fatty acids (ω-3 PUFAs) has been positively associated with a number of health benefits including prevention and reduction of cardiovascular diseases, inflammation and cancers. However, little is known about the impact of ω-3 PUFAs on liver fibrosis. In this study, we used CCl_4_-induced liver fibrosis mouse model and found that YAP/TAZ is over-expressed in the fibrotic liver and activated HSCs. Fish oil administration to the model mouse attenuates CCl_4_-induced liver fibrosis. Further study revealed that ω-3 PUFAs down-regulate the expression of pro-fibrogenic genes in activated HSCs and fibrotic liver, and the down-regulation is mediated via YAP, thus identifying YAP as a target of ω-3 PUFAs. Moreover, ω-3 PUFAs promote YAP/TAZ degradation in a proteasome-dependent manner. Our data have identified a mechanism of ω-3 PUFAs in ameliorating liver fibrosis.

As one of the most common consequences of chronic liver diseases, liver fibrosis represents a significant world-wide health problem. It can be classified as a wound-healing response to chronic hepatic injury, which may be caused by alcohol abuse, hepatitis virus infection, autoimmune disorders, biliary obstruction or nonalcoholic steatohepatitis, and is characterized by excessive scar formation due to overproduction and deposition of extracellular matrix (ECM) components resulted from an imbalance of ECM molecule metabolism either by an increased synthesis or decreased degradation of ECM components or both^1^,^2^,^3^. Activation of hepatic stellate cells (HSCs), a major cell type responsible for increased synthesis of ECM proteins, represents a crucial event in the pathogenic sequence of fibrosis and thus provides an important framework to define potential strategies for anti-fibrotic therapy. Like liver sinusoidal endothelial cells and Kupffer cells, quiescent HSCs are non-parenchymal cells. They normally reside in the space of Disse, containing bunches of vitamin A-riching lipid droplets, while activated HSCs lose cytoplasmic lipid droplets and trans-differentiate to proliferative, fibrogenicmyofibroblasts, and are characterized by the over-expression of α-SMA^1^,^2^,^3^,^4^.

Fish oil supplementation, usually containing a mix of eicosapentaenoic acid (EPA) and docosahexaenoic acid (DHA) as major constituents, has been reported to be generally beneficial in the onset and progression of several chronic diseases, including coronary artery disease and atherosclerosis, diabetes and cancer^5^,^6^,^7^,^8^,^9^,^10^. Recently, there has been growing interest in ω-3 PUFAs supplementation as potential treatment for liver diseases^11^,^12^,^13^,^14^. However, there is little information regarding the impact of ω-3 PUFAs on the progression of liver fibrosis. Interestingly, EPA and DHA exert a potent anti-oxidative stress and anti-inflammatory activity in various cell lines, suggesting that ω-3 PUFAs may have an anti-fibrotic effect on the liver.

Elevated levels of the transcriptional regulators Yes-associated protein (YAP) and transcriptional coactivators with PDZ-binding motif (TAZ), key effectors of the Hippo pathway, are associated with a broad range of aggressive cancers including hepatocellular carcinoma^15^,^16^,^17^. Moreover, YAP/TAZ has also been shown to play an essential role in controlling liver cell fate and regulating liver response to cholestasis^18^. In addition, a large body of data has accumulated showing that CTGF, the most highly characterized YAP target gene^19^, is over-expressed in fibrotic liver and activated HSCs. CTGF induces the synthesis and secretion of ECM proteins, notably of fibrillar collagens which are a major component of fibrous deposits^20^,^21^,^22^,^23^,^24^. On the other hand, transforming growth factor-β (TGF-β) has been considered the most important factor that drives liver fibrosis. Suppression of TGF-β expression or its downstream signaling pathway can ameliorate or even prevent liver fibrosis^25^. Interestingly, it has been reported that YAP/TAZ, interact with TGF-β-induced SMAD2/3 in the nucleus, suggesting that YAP/TAZ·TEAD·SMAD2/3 complexes coordinately regulate TGF-β-induced transcriptional program^16^,^26^. Furthermore, studies have shown that Notch signaling participates in the progression of fibrosis and can directly up-regulate Col1α1 and Col1α2 promoter activity through a Hes1-dependent mechanism^27^. More recently, the Notch pathway, including Notch1/2, Jag1 and the Notch target genes Hes1 and Sox9 have also been shown to be directly targeted by the YAP/TEAD complex^18^. Similarly, it has also been reported that YAP/TAZ functions as a regulator of microprocessor activity and regulates biogenesis of miRNA^28^,^29^,^30^, some of which play an important role in liver fibrosis. Moreover, recent study has shown that YAP drives the earliest changes in gene expression during hepatic stellate cell activation^31^. Elevated YAP/TAZ expression correlates with bile duct proliferation and fibrosis^15^,^32^,^33^. Connecting these previously reported phenomena, we focused on the transcriptional effectors YAP/TAZ as a potential regulator of liver fibrosis.

In this study, we demonstrate for the first time that ω-3 PUFAs attenuate CCl_4_-induced liver fibrosis *in vivo* and inhibit hepatic stellate cell proliferation and activation. Our findings indicate that the expression of YAP and TAZ are up-regulated in CCl_4_-induced liver fibrosis. Furthermore, ω-3 PUFAs inhibit hepatic stellate cell lines proliferation and activation by promoting YAP/TAZ degradation.

## Results

### Fish oil attenuates CCl_4_-induced liver fibrosis

**Figure 1:**
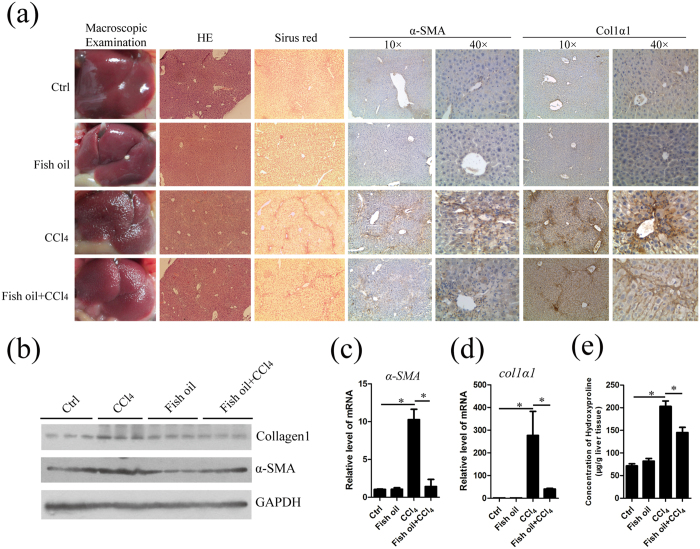
Fish oil attenuates CCl_4_-induced liver fibrosis *in vivo.* Mice were treated with normal diet (control), fish oil diet (fish oil), injected with CCl_4_ (CCl_4_) or injected with CCl_4_ combined with fish oil diet treatment (fish oil/CCl_4_) on postnatal week 8. Liver tissues were used for analysis. (**a**) The liver histology was examined by means of macroscopic morphology, HE staining and sirius red staining. *α-SM*A and collagen1 signals were visualized by immunohistochemistry. (**b**) α-SMA and collagen1 signals were examined and quantified by western blot. GAPDH served as the loading control. The gels were cropped and the full-length gels are presented in Supplementary Fig. 5. (**c**,**d**) Total RNA from liver tissue in control, fish oil, CCl_4_ and fish oil/CCl_4_ mice was isolated and subsequently used for the detection of the mRNA of *α-SMA* (**c**) and *collagen1α1* (**d**) by real-time PCR analysis. (**e**) The content of hydroxyproline was detected by commercially available hydroxyproline detection kits. The data are expressed as the mean ± SEM for triplicate experiments. *P < 0.05.

There has been growing interest in ω-3 PUFAs supplementation as potential treatment for liver diseases^11^,^12^,^13^,^14^. However, there is little information regarding the impact of ω-3 PUFAs on the progression of liver fibrosis. Therefore, we examined the potency of fish oil in attenuating hepatic fibrosis in mice treated with CCl_4_ 34,35,36. Firstly, we measured detailed composition of fatty acids in liver tissues by using gas chromatography and found that the ω-6 PUFAs (18:2 n-6, 20:4 n-6, and 22:4 n-6) content (% of total fatty acids) decreased and the ω-3 PUFAs (20:5 n-3, 22:5 n-3, and 22:6 n-3) content (% of total fatty acids) increased in the fish oil group and fish oil/CCl_4_ group (Supplemental Table 1). Figure 1a shows the gross appearance of representative livers from animals treated with vehicle, fish oil, CCl_4_ alone or CCl_4_ in combination with a medium dose of fish oil. There were fewer nodules on hepatic surfaces in the fish oil/CCl_4_ group compared to CCl_4_ group, indicating lower degree of fibrosis. HE staining shows for gross morphology and the sirius red staining for the determination of specific fibrotic regions in the liver tissues. Administration of CCl_4_ for 8 weeks induced a high level of collagen deposition in fibrotic septa between nodules in hepatocytes. Fish oil administration reduced the histological signs of collagen fiber percentages. α-SMA is a good marker for the activation of HSCs during fibrosis and reactivity, principally in fibrous septa and regenerative nodules in fibrotic livers. Immunohistochemical staining revealed that CCl_4_-treated liver exhibited increased expression of α-SMA and collagen1 and only faint traces of α-SMA and collagen1-positive cells were detected in fish oil/CCl_4_ group. The result was further confirmed by western blot (Fig. 1b) and real time-PCR (Fig. 1c,d). Furthermore, the hydroxyproline content, a modified amino acid uniquely found in a high percentage in collagen, showed an approximate 30% reduction in fish oil/CCl_4_ group compared with CCl_4_ group (Fig. 1e), suggesting that fish oil could ameliorate the degree of liver fibrosis. In addition, serum alanine aminotransferases (ALT) and aspartate aminotransferases (AST), which can be released from liver tissue into the circulation in proportion to the degree of hepatocellular damage, were decreased in fish oil/CCl_4_ group relative to CCl_4_ group (Supplemental Table 2). Taken together, dietary supplementation with fish oil protects against CCl_4_-induced liver fibrosis.

### ω-3 PUFAs inhibit the proliferation of hepatic stellate cells

**Figure 2:**
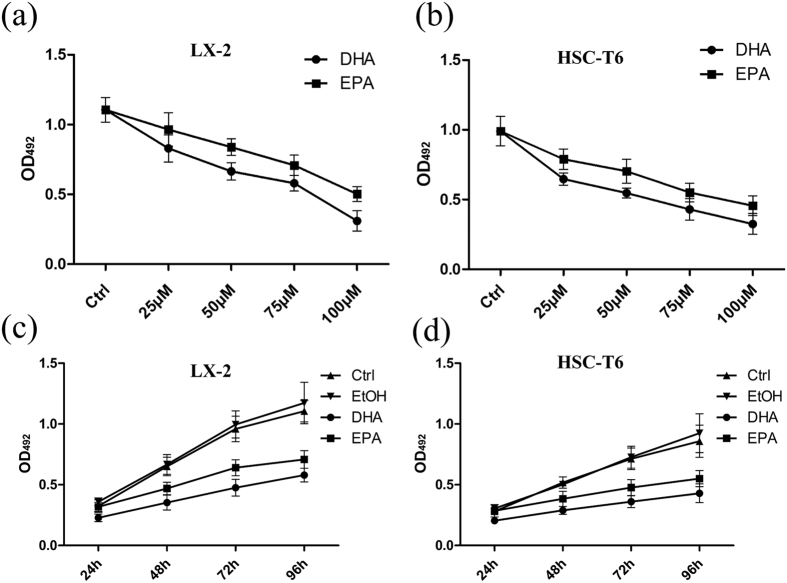
DHA and EPA inhibit the proliferation of LX-2 and HSC-T6 cells. (**a**,**b**) LX-2 (**a**) and HSC-T6 (**b**) cells were assessed by an MTT assay for viability following exposure for 72 h to media containing 10% FBS and varying concentrations of DHA or EPA. (**c**,**d**) LX-2 (**c**) and HSC-T6 (**d**) cells were assessed by an MTT assay for viability following exposure for 24 h, 48 h, 72 h and 96 h, respectively to media containing 10% FBS and 75 μM DHA or EPA. The data are expressed as the mean ± SEM for triplicate experiments. *P < 0.05.

Hepatic fibrosis is characterized by excessive deposition of ECM proteins. HSCs undergo rapid activation, functional and morphological changes in response to hepatic injury, and become a major source of ECM production^1^,^3^. Sustained activation involves several discrete changes in cell behavior, such as proliferation, chemotaxis and fibrogenesis^1^,^3^. Firstly, we investigated the effect of fish oil intake on the proliferation of HSCs in the animal model. Cell counting of the HSCs immediately isolated from liver tissues showed that the amount of these cells in fish oil/CCl_4_ group was significantly reduced than that in CCl_4_ group. However, the amount of HSCs in control mice was not reduced by fish oil (Supplemental Table 3), suggesting that fish oil inhibits the proliferation of the activated HSCs *in vivo*. Furthermore, the inhibitory effect of fish oil on the proliferation of HSCs in fibrotic liver tissues was also demonstrated by IHC for PCNA. The amount of PCNA positive cells in fish oil/CCl_4_ group was reduced compared with that in CCl_4_ group (Supplemental Fig. 1a). In addition, the result of qRT-PCR showed the mRNA levels of *Ki67* and *PCNA* in immediately isolated HSCs were also decreased in fish oil/CCl_4_ group compared with that in CCl_4_ group (Supplemental Fig. 1b). To confirm the anti-fibrotic effects of dietary fish oil observed *in vivo*, we investigated the effect of DHA and EPA, the two primary ω-3 PUFAs in fish oil, on the proliferation of cultured HSCs including LX-2 and HSC-T6 cells by MTT assay. Firstly, we used increasing amounts up to 100 μM DHA or EPA to treat LX-2 and HSC-T6 cells for 6 h, respectively, to evaluate whether DHA or EPA has a toxic effect. Trypan blue staining showed no difference in the cells treated with either DHA or EPA compared with the control cells (Supplemental Fig. 2a,b), suggesting that the concentrations of DHA or EPA used in the present study caused no toxic effect on LX-2 and HSC-T6 cells. The data of MTT assay revealed remarkable attenuated proliferation when LX-2 and HSC-T6 cells were treated with different concentration of DHA or EPA for 72 hours, with the maximal effect at 100 μM (Fig. 2a,b). It indicates that DHA and EPA are capable of inhibiting the proliferation of LX-2 and HSC-T6 cells in a dose-dependent manner. In addition, treatment with 75 μM DHA or EPA for varying durations on LX-2 and HSC-T6 cells demonstrated that the cell viabilities declined continuously with the prolonged treatment of DHA or EPA relative to control, indicating a time-dependent suppression of cell proliferation (Fig. 2c,d). Taken together, these results indicate that ω-3 PUFAs significantly suppress the proliferation of activated HSCs.

### ω-3 PUFAs down-regulate the expression of pro-fibrogenic genes in hepatic stellate cells

**Figure 3:**
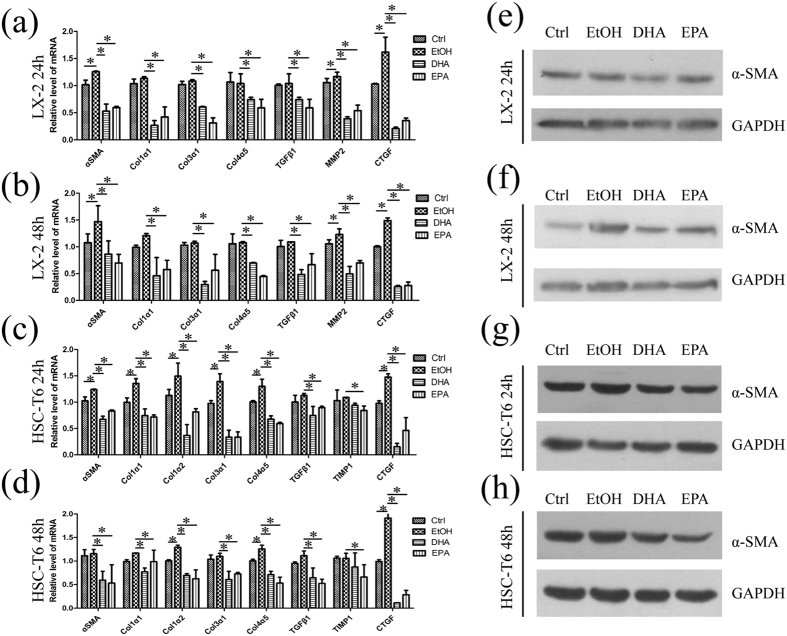
DHA and EPA down-regulate pro-fibrogenic genes in LX-2 and HSC-T6 cells. (**a**–**d**) LX-2 (**a**,**b**) and HSC-T6 (**c**,**d**) cells were exposed for 24 h or 48 h to media containing 10% fetal bovine serum, ethanol, 75 μM DHA or EPA and then total RNA was isolated. The expression of *α-SMA, collagen1α1, collagen1α2, collagen3α1, collagen4α5, mmp2, tgfβ1, timp1* and *ctgf* were measured by qPCR. (**e**–**h**) With the same treatment in (**a**–**d**) total protein of LX-2 (**e**,**f**) and HSC-T6 (**g**,**h**) cells were isolated and α-SMA was measured and quantified by western blot. GAPDH served as the loading control. The gels were cropped and the full-length gels are presented in Supplementary Fig. 5. The data are expressed as the mean ± SEM for triplicate experiments. *P < 0.05.

HSCs generate fibrosis not only by increasing cell number, but also by increasing matrix production through regulated changes in gene expression, which reflect the convergence of several intracellular signaling cascades on regulatory regions of key molecular signals^37^. Therefore, we investigated the effects of DHA and EPA on the expression of pro-fibrogenic genes in HSC lines. Quantitative PCR analysis showed that ethanol, the solvent of DHA and EPA, increased the expression of *α-SMA* and *ctgf* in LX-2 cells, while it up-regulated *α-SMA, collagen1α1, collagen1α2, collagen3α1, collagen4α5* and *ctgf* mRNA levels in HSC-T6 cells, suggesting that ethanol is involved in liver fibrosis (Fig. 3a–d). Treatment with 75 μM DHA down-regulated the mRNA level of pro-fibrogenic genes, including *α-SMA, collagen1α1, collagen1α2, collagen3α1, collagen4α5, mmp2* and *ctgf* in LX-2 cells, relative to ethanol treatment, with a significant decrease of *ctgf* by reduction to 13% at 24 h (Fig. 3a,b). Besides, the modulation of pro-fibrogenic genes by 75 μM EPA showed similar trends in LX-2 cells. Interestingly, hepatic content of *tgf-β1*, a key pro-fibrogenic cytokine that regulates HSC activation and synthesis of ECM, was also reduced by DHA or EPA in LX-2 and HSC-T6 cells. Similar to the results in LX-2, 75 μM ω-3 PUFAs treatment for 24 h and 48 h also decreased pro-fibrogenic genes expression in HSC-T6 cells (Fig. 3c,d). Western blot for α-SMA also presented a decreased level in DHA or EPA treated LX-2 and HSC-T6 cells for 24 h and 48 h (Fig. 3e–h).

### Fish oil down-regulates the hepatic expression of fibrogenic genes induced by CCl_4_

**Figure 4:**
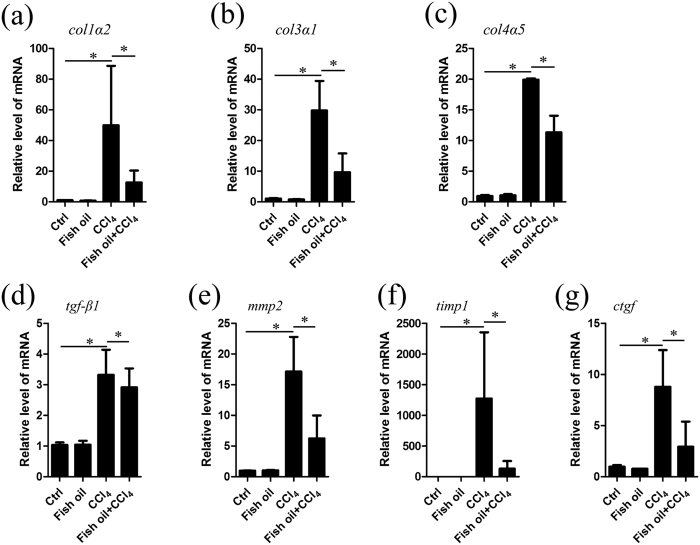
Fish oil down-regulates the expression of fibrogenic genes induced by CCl_4._ (**a**–**g**) Total RNA of liver tissue in control, fish oil, CCl_4_ and fish oil/CCl_4_ mice were isolated. The following fibrogenic genes, including *collagen 1α2* (**a**), *collagen 3α1* (**b**)*, collagen4α5* (**c**), *tgf-β1* (**d**), *mmp2* (**e**), *timp1* (**f**) and *ctgf* (**g**) were assayed by real-time PCR. The data are expressed as the mean ± SEM for triplicate experiments. *P < 0.05.

Stellate cell initiation promotes changes in gene expression and phenotype that render the cells susceptible to the changing environment and stimuli in the injured liver^1^,^3^,^37^. Given the evidence that ω-3 PUFAs down-regulate the expression of pro-fibrogenic genes in cultured HSCs, we further examined whether fish oil affects the expression of fibrogenic genes in fibrotic livers. Treatment with CCl_4_ significantly increased the mRNA levels of factors involved in fibrillar ECM synthesis, such as *collagen1α1* (Fig. 1d)*, collagen1α2, collagen3α1, collagen4α5, mmp2, timp1, tgf-β1,* and *ctgf* (Fig. 4a–g) relative to control group. Further analysis revealed that expression of genes involved in fibrogenesis including *collagen1α1* (86% reduction), *collagen1α2* (70% reduction), *collagen3α1* (67% reduction), *collagen4α5* (45% reduction), *mmp2* (65% reduction), *timp1* (91% reduction) and *ctgf* (62% reduction), were reduced by fish oil treatment (Fig. 4). In addition, we used the immediately isolated HSCs from the mouse model to investigate the expression of these pro-fibrogenic genes and found the results (Supplemental Fig. 3) are consistent with those obtained in cultured HSCs (Fig. 3a–h) and in liver tissues (Fig. 4). Taken together, fish oil down-regulates the expression of fibrogenic genes induced by CCl_4_.

### YAP/TAZ is over-expressed in fibrotic liver and fish oil reduces the protein level of YAP/TAZ

**Figure 5:**
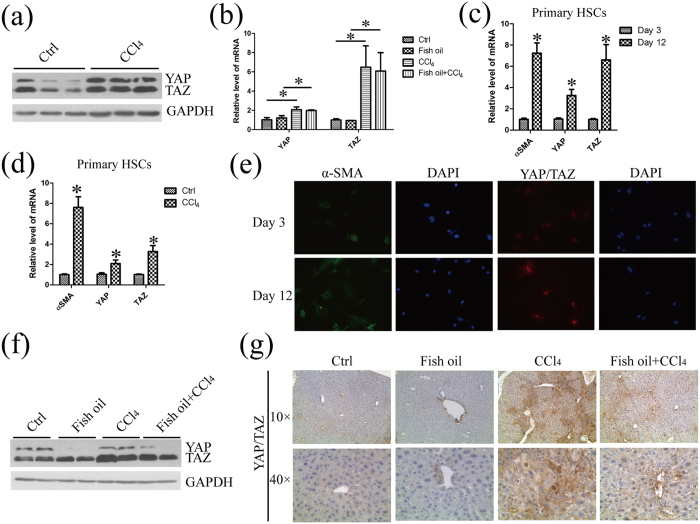
YAP/TAZ are over-expressed in fibrotic liver and fish oil decreases the protein level of YAP/TAZ without changing their mRNA level in liver tissue. (**a**) Total protein of liver tissue in control mice and CCl_4_-treated mice were prepared and subjected to western blot analysis with the specified antibodies to YAP/TAZ. GAPDH served as the loading control. The gels were cropped and the full-length gels are presented in Supplementary Fig. 5. (**b**) Total RNA of liver tissue prepared from mice of the indicated groups was used for the detection of Yap/Taz mRNA by real-time PCR analysis. (**c**) The total RNA was extracted from primary freshly isolated quiescent HSCs at day 3 and in vitro activated HSCs at day 12 and was subsequently used for the detection of *α-SMA* and *Yap/Taz* mRNA by real-time PCR analysis. (**d**) The total RNA extracted from primary freshly isolated quiescent HSCs at day 3 from healthy and fibrosis mice was applied for detection of *α-SMA* and *Yap/Taz* mRNA by real-time PCR. (**e**) Primary freshly isolated quiescent HSCs at day 3 and *in vitro* activated HSCs at day 12 were stained with antibodies against the α-SMA and YAP/TAZ by immunofluorescence. The DAPI staining (blue) was used to verify cell nucleus. (**f**) YAP/TAZ signaling in the liver tissue of indicated mice were examined and quantified by western blot. GAPDH served as the loading control. The gels were cropped and the full-length gels are presented in Supplementary Fig. 5. (**g**) Liver sections from mice of the indicated genotypes were immunostained and quantified with anti-YAP/TAZ antibody. The data are expressed as the mean ± SEM for triplicate experiments. *P < 0.05.

Elevated levels of the transcriptional regulators YAP and TAZ, key effectors of the Hippo pathway, are associated with hepatocellular carcinoma and various liver diseases^15^,^17^. More recently, YAP/TAZ has also been shown to play essential roles in controlling liver cell fate and HSC activation^18^. To confirm whether YAP/TAZ is associated with liver fibrosis, we firstly analyzed YAP /TAZ expression in liver tissues from normal mice and fibrotic mice. As the results shown in Fig. 5a, the protein level of YAP/TAZ was much higher in fibrotic liver tissues, compared with those in normal liver tissues. Moreover, CCl_4_ significantly increased the mRNA level of *Yap/Taz* by 2.3 fold and 5.7 fold, respectively, suggesting that YAP/TAZ is over-expressed in fibrotic livers (Fig. 5b). The expression of *α-SMA* in seeded HSCs was increased in day 12, indicating that HSCs were activated *in vitro*. The mRNA level of *Yap/Taz* was also increased in the same HSCs in day 12, compared with that in quiescent cells in day 3 (Fig. 5c). In addition, we performed qPCR in HSCs isolated from healthy and fibrosis mice. The mRNA levels of *α-SMA, Yap/Taz* are up-regulated in HSCs isolated from fibrosis mice (Fig. 5d). Moreover, the results of immunofluorescence revealed that the majority of HSCs exhibit increased staining of cytoplasmic α-SMA and nuclear YAP/TAZ after 12 days *in vitro* culture, relative to that in day 3 (Fig. 5e). All these data revealed that YAP/TAZ is over-expressed in fibrotic liver and activated HSCs. Furthermore, we also performed qPCR and western blot to validate the association between ω-3 PUFAs and YAP/TAZ. Although CCl_4_ significantly increased the mRNA level of *Yap/Taz*, fish oil has no effect on the mRNA level of *Yap/Taz* in fish oil/CCl_4_ group, compared with CCl_4_ group (Fig. 5b). Interestingly, the protein level of YAP/TAZ was decreased 80% and 30%, respectively, by fish oil in fish oil/CCl_4_ group relative to CCl_4_ group (Fig. 5f). The inhibitory effect of fish oil on YAP/TAZ over-expression in fibrotic liver tissues was also demonstrated by IHC (Fig. 5g). Thus, we conclude that ω-3 PUFAs decrease the protein level but not the mRNA level of YAP/TAZ in CCl_4_-induced fibrotic livers.

**Figure 6:**
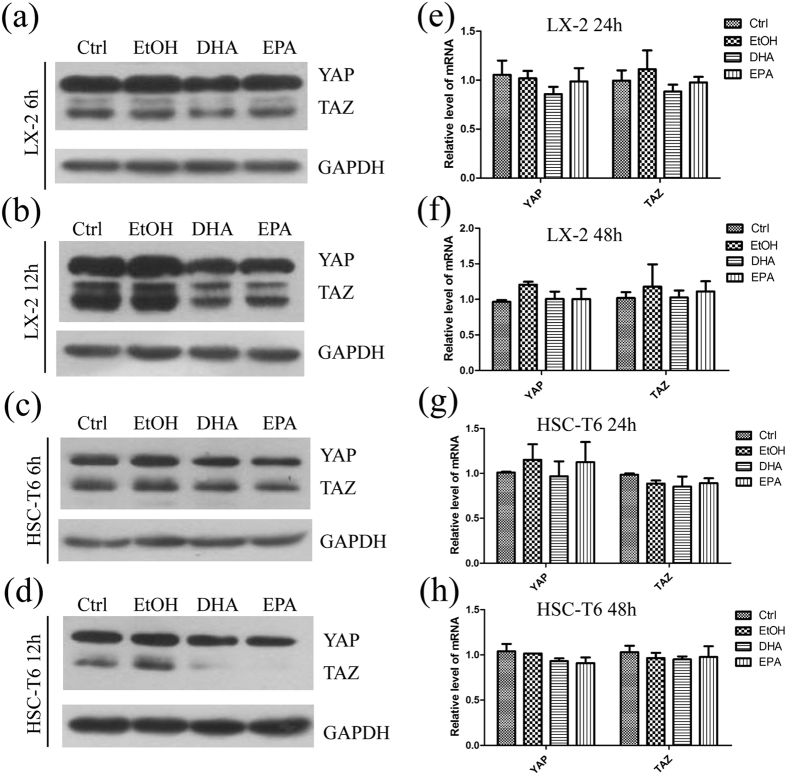
DHA and EPA decrease the protein level but not the mRNA level of YAP/TAZ in LX-2 and HSC-T6 cells. (**a**–**d**) LX-2 (**a**,**b**) and HSC-T6 (**c**,**d**) cells were exposed for 6 or 12 h to media containing 10% fetal bovine serum, ethanol, 75 μM DHA or EPA. Cell lysates from both cell lines were subsequently assessed by immunoblot to determine the protein levels of YAP/TAZ. GAPDH was used as internal control. The gels were cropped and the full-length gels are presented in Supplementary Fig. 6. (**e**–**h**) The total RNA were extracted from LX-2 (**e**,**f**) and HSC-T6 (**g**,**h**) cells exposed for 24 h or 48 h to media containing 10% fetal bovine serum, ethanol, 75 μM DHA or EPA. The extracted RNA was subsequently used for qPCR. The data are expressed as the mean ± SEM for triplicate experiments. *P < 0.05.

Based on the finding above, we also examined whether ω-3 PUFAs regulate the expression of YAP/TAZ in LX-2 and HSC-T6 cells. The protein level of YAP/TAZ decreased significantly in LX-2 (Fig. 6a,b; Supplemental Fig. 4a,b) and HSC-T6 (Fig. 6c,d; Supplemental Fig. 4c,d) cells with the treatment of DHA or EPA for 6 h, 12 h, 24 h or 48 h, relative to ethanol treatment. Interestingly, no significant reduction of *Yap/Taz* mRNA was observed upon 75 μM DHA or EPA treatment (Fig. 6e–h). In conclusion, these findings indicate that DHA and EPA reduce YAP/TAZ protein level with no change at mRNA level in HSCs, which was consistent with the findings in fibrotic livers (Fig. 5b).

### ω-3 PUFAs down-regulate the expression of pro-fibrogenic genes via YAP

**Figure 7:**
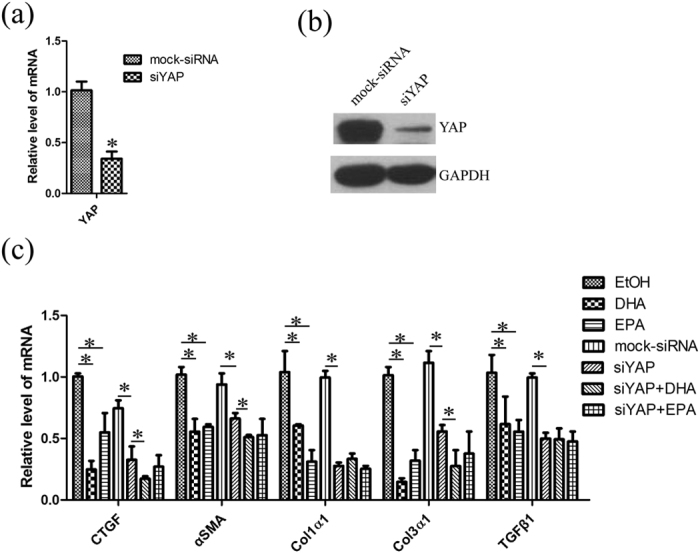
DHA and EPA inhibit the expression of pro-fibrogenic genes via YAP in LX-2. (**a**,**b**) LX-2 cells were transfected with YAP siRNA for 48 h and then the mRNA and protein were prepared for qPCR analysis (**a**) and western blot (**b**). The gels were cropped and the full-length gels are presented in Supplementary Fig. 6. (**c**) 48 hours after transfection with YAP siRNA, LX-2 cells were treated with ethanol, 75 μM DHA or EPA for additional 24 h. The total RNA was extracted and used for qPCR analysis of the representative panel of pro-fibrogenic genes. The data are expressed as the mean ± SEM for triplicate experiments. *P < 0.05.

We then sought to determine whether YAP plays a role in down-regulating the expression of pro-fibrogenic genes by ω-3 PUFAs. LX-2 cells were transfected with YAP specific siRNA, and the mRNA of YAP could be effectively knocked down by RNA interfering, resulting in reduced level of this protein (Fig. 7a,b). The expression of pro-fibrogenic genes in LX-2 cells, including *α-SMA, collagen 1α1, collagen 3α1, tgf-β1* and *ctgf,* were analysed with qPCR and the results showed levels of mRNA were decreased when the cells were treated with DHA or EPA, relative to ethanol treatment (Fig. 7c). However, the suppressive effect of DHA and EPA on the pro-fibrogenic genes expression did not strengthen further when YAP expression has been silenced with specific siRNA (Fig. 7c), indicating that ω-3 PUFAs inhibit expression of pro-fibrogenic genes via YAP in LX-2 cells.

### ω-3 PUFAs promote the proteasome-mediated degradation of YAP/TAZ

**Figure 8:**
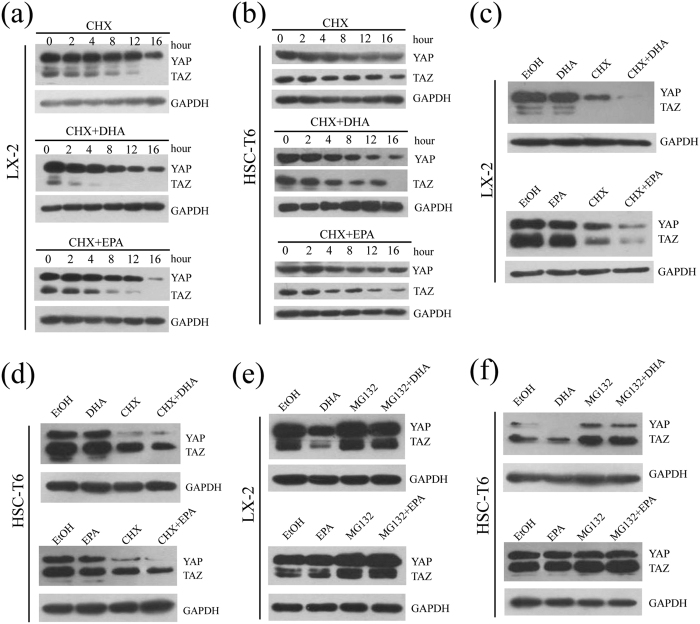
DHA and EPA induce YAP/TAZ degradation in LX-2 cells through proteasome-dependent pathway. (**a**,**b**) LX-2 (**a**) and HSC-T6 (**b**) cells cultured in medium containing 10% FBS were treated with CHX alone, CHX in combination with 75 μM either DHA or EPA for the indicated durations. Cell lysates were prepared and used for immunoblot and GAPDH was used as a loading control. (**c**,**d**) LX-2 (**c**) and HSC-T6 (**d**) cells cultured in medium containing 10% FBS were treated with ethanol, 75 μM DHA or EPA, CHX, CHX in combination with 75 μM DHA or EPA for 12 h. Cell lysates were prepared and used for immunoblot and GAPDH was used as a loading control. (**e**,**f**) LX-2 (**e**) and HSC-T6 (**f**) cells were treated with ethanol, 75 μM DHA or EPA, MG132, MG132 in combination with 75 μM DHA or EPA for 24 h and were subsequently lysed for immunoblot. The gels were cropped and the full-length gels are presented in Supplementary Fig. 7. The data are expressed as the mean ± SEM for triplicate experiments. *P < 0.05.

To further determine the discrepant effects of ω-3 PUFAs on the mRNA and protein expression level of YAP/TAZ, we firstly investigated whether it is ascribable to blockage of protein synthesis. The protein translation inhibitor cycloheximide (CHX) was therefore applied to treat LX-2 and HSC-T6 cells, and under conditions of no protein translation, it was demonstrated that YAP protein decreased in a time-dependent manner, showing a half-life of 16 h (t_1⁄2_ = 16 h) in both cell lines. Furthermore, the reduction in the YAP protein was more pronounced to 8 h with DHA or EPA in the presence of CHX (t_1⁄2_ = 8 h) (Fig. 8a,b). For TAZ, more rapid degradation of the protein could be observed in LX-2 (t_1⁄2_ = 4 h) and HSC-T6 cells (t_1⁄2_ = 8 h) treated with CHX. In addition, DHA or EPA, in the presence of CHX, greatly destabilize TAZ in LX-2 (t_1⁄2_  = 2 h) and HSC-T6 cells (t_1⁄2_  = 4 h) (Fig. 8a,b). Additionally, at the time point of 12 h, there were significant additive effects between ω-3 PUFAs and CHX when LX-2 and HSC-T6 cells were treated with each drug (Fig. 8c,d). The additive reduction of the YAP/TAZ protein levels by addition of ω-3 PUFAs beyond that already elicited by CHX suggested that ω-3 PUFAs-induced reduction in YAP/TAZ protein levels is not due to blockage of protein translation.

Given the evidence that ω-3 PUFAs reduce the protein level of YAP/TAZ, but have no effect on protein translation, we next investigated whether ω-3 PUFAs affect the stability of YAP/TAZ. LX-2 and HSC-T6 cells were treated with MG132, a proteasome inhibitor, to prevent proteasome-dependent degradation. As shown in Fig. 8e, treatment with MG132 increased the YAP/TAZ protein levels compared with the control, suggesting that YAP/TAZ is degraded by the proteasome. Conspicuously, the combined treatment of MG132 and ω-3 PUFAs significantly increased the protein levels of YAP/TAZ compared with ω-3 PUFAs treatment alone. These results suggest that ω-3 PUFAs decreased the cellular levels of YAP/TAZ through a proteasome-dependent process (Fig. 8e,f).

## Discussion

Hepatic fibrosis is the final common pathway for all forms of chronic liver disease, including alcohol abuse, hepatitis virus infection, autoimmune disorders, biliary obstruction or nonalcoholic steatohepatitis^2^,^3^. Several agents, such as CCl_4_, TAA and DEN have long been used to induce animal model for liver fibrosis^1^,^38^. CCl_4_-induced liver fibrosis has been extensively used in mice because hepatic responses to chronic CCl_4_ stimulation in mice are shown to be greatly similar to human cirrhosis^39^.

The dietary intake of the long chain ω-3 PUFAs, especially EPA and DHA, has been positively associated with a number of health benefits including prevention and reduction of cardiovascular diseases, some inflammatory diseases as well as some cancers^6^,^7^,^40^. Previous studies have shown that ω-3 PUFAs exert a potent anti-inflammatory activity and anti-fibrosis effect in the models of fibrosis including pulmonary and cardiac fibrosis^5^,^41^. However, little is known about the association between the ω-3 PUFAs and liver fibrosis. In this study, our results suggest that fish oil greatly improves the state of liver fibrosis, as measured by macroscopic examination, HE staining, Sirius red staining and IHC for α-SMA and type 1 collagen. Furthermore, quantitative analysis of hydroxyproline levels showed an approximate 30% reduction in fish oil/CCl_4_ group compared with CCl_4_ group. Our data also reveal that fish oil also decreases the expression of pro-fibrotic genes such as *collagen1α1, collagen1α2, collagen3α1, collagen 4α5, mmp2, timp1* and *ctgf*. Altogether, our results demonstrate an anti-fibrotic effect of ω-3 PUFAs in liver *in vivo*.

The key event in the development of liver fibrosis is the activation of hepatic stellate cell^1^. HSC activation, the transdifferentiation of quiescent HSCs to myofibroblast like, collagen-producing HSCs, is a major phenomenon in the initiation and progression of liver fibrosis^1^,^4^. As a consequence, one strategy for reducing fibrosis is to prevent the activation of HSCs. Both the LX-2 cell line (human HSC line) and the HSC-T6 cell line (rat HSC line) have been widely used in fibrosis research because they are more amenable to high level transfection of plasmid than primary HSCs. In addition, the phenotype of both HSC lines is most similar to that of “activated” cells *in vivo*. These cells express α-SMA, desmin, glial acidic fibrillary protein (GFAP) and matrix metalloproteinase under all culture conditions^1^,^4^,^37^,^42^.

In this study, we investigate the effect of ω-3 PUFAs in these cell lines. It has been reported that ethanol induced the production of collagen1α1 and α-SMA in human or mouse HSCs^23^. Since the DHA and EPA used in this study were diluted in ethanol, we also investigate the effect of ethanol in these two HSC cell lines. As the results shown in the text, DHA and EPA significantly suppressed LX-2 and HSC-T6 cell viability in a dose and time-dependent manner. Furthermore, consistent with the results in CCl_4_-induced liver fibrosis, DHA and EPA down-regulated the mRNA level of pro-fibrogenic genes in LX-2 and HSC-T6 cells. All the results indicated that DHA and EPA inhibit HSC activation which partly related to regulating proliferation and eventually reduce fibrogenesis. In addition, our study *in vitro* showed that ethanol increases the expression of *α-SMA* and *ctgf* in LX-2, while in HSC-T6, the expression of *α-SMA, collagen1α1, collagen1α2, collagen3α1, collagen4α5* and *ctgf* was up-regulated by ethanol. The reason for this discrepancy may be that the action ways of ethanol are various in cell lines of different species and backgrounds. Furthermore, we found that *ctgf* has the highest depression changes induced by DHA and EPA, suggesting that the down-regulation of the mRNA level of pro-fibrotic genes may be dependent on CTGF. As CTGF is the most highly characterized YAP/TAZ target gene, YAP/TAZ may mediate the decrease in the expression of CTGF by DHA and EPA.

The Hippo pathway has been demonstrated as an important regulator for liver development^43^. For example, liver specific over-expression of *Yap*, a Hippo pathway effector, in the mouse resulted in *Yap*-hyperactivity causing hepatomegaly^44^,^45^. Activation of HSCs is a general response to liver damage inducing the over-production of ECM to protect the damaged tissue. It has been shown that YAP is an early and key regulator in the HSC activation process^31^. A function of YAP as a stress sensor and driver of regenerative behavior could be observed in HSCs. Furthermore, fibroblasts expressing active YAP promote fibrosis when transplanted into murine lungs, demonstrating that YAP/TAZ activation can drive a pro-fibrotic response *in vivo*^46^. Therefore, inhibition of the YAP/TAZ activity could reduce fibrogenesis.

In the current study, to investigate whether ω-3 PUFAs inhibit HSCs activation and proliferation through YAP/TAZ, we firstly explore the expression of YAP/TAZ in liver fibrosis. We found that YAP/TAZ was over-expressed in fibrotic liver tissue and ω-3 PUFAs decreased the protein level but not the mRNA level of YAP/TAZ in liver tissue and HSC lines. Interestingly, YAP expressed higher than TAZ in LX-2 cell lines. Moreover, knockdown of YAP abolished the mRNA reduction of pro-fibrotic genes such as *ctgf, α-SMA, collagen1α1, collagen3α1* and *tgf-β1* induced by DHA and EPA, suggesting that ω-3 PUFAs inhibit HSC lines activation through YAP. Our data have demonstrated that decrease of YAP/TAZ induced by DHA and EPA was not due to decreased transcription of gene, since the levels of mRNA were not affected by ω-3 PUFAs treatment in both cell types. All the results suggest that the down-regulation of YAP/TAZ may be occurred in the post transcriptional level. In our *in vitro* study, we found that the half-lives of YAP and TAZ were markedly reduced whenLX-2 and HSC-T6 cells were incubated with DHA and EPA under the condition of blocked translation. This finding confirmed that down-regulation of YAP/TAZ by ω-3 PUFAs occurred in the post transcriptional level. We also observed that ω-3 PUFAs down-regulated the protein level of YAP/TAZ as early as 6 hours, and this result suggest that inhibition of YAP/TAZ could prevent the fibrogenesis at an early stage because YAP activation and the subsequent induction of target genes expression are early events during HSC activation^31^. Furthermore, the application of MG132, a proteasome inhibitor, in combination with DHA or EPA to treat the cells revealed that ω-3 PUFAs decrease the cellular levels of YAP/TAZ through a ubiquitin-proteasome-dependent process.

Our results have shown that ω-3 PUFAs reduce YAP/TAZ protein levels in fibrotic liver over-expressing these proteins, the mechanism how YAP/TAZ is up-regulated still needs further studies. Additionally, the mechanism of YAP/TAZ to regulate profibrotic genes is also unknown. It is possible that TAZ/YAP and transcription factor(s) complexes coordinately regulate the expression of profibrotic genes. In conclusion, using different HSCs and animal model, we have shown that ω-3 PUFAs ameliorate CCl_4_-induced liver fibrosis *in vivo* and inhibit HSCs proliferation and activation via promoting YAP/TAZ degradation, which are over-expressed in fibrotic liver. According to our current results, ω-3 PUFAs may have a beneficial role in the treatment of chronic liver diseases caused by ongoing hepatic damage.

## Methods

### Cell lines and fatty acid treatment

LX-2 cells, an immortalized human HSCs line, were obtained from Merck Millipore (Beijing, China). A rat HSC line (HSC-T6) was obtained from the cell bank of the Chinese Academy of Sciences (Shanghai, China). For all experiments, the cells were subjected to no more than 20 cell passages. Both cell lines were maintained in DMEM medium (Gibco, Gaithersburg, MD,USA) supplemented with 10% fetal bovine serum (Gibco, Gaithersburg, MD,USA) at 37 °C and in an atmosphere of 5% CO_2_. All culture media contained 100 U/ml of penicillin and 100 μg/ml of streptomycin. Stock solutions of DHA and EPA in ethanol were stored at −20 °C and diluted in complete growth medium before experiments (final concentration of ethanol, 0.1% v/v). Cells were treated with DHA or EPA for 24 or 48 hours at concentration of 75 μM in complete growth medium.

### Cell proliferation assay

Cell growth was assessed by MTT (Sigma-Aldrich, St. Louis, MO, USA) dye conversion at 492 nm following manufacturer’s instructions. Briefly, Cells (5 × 10^3^/well) were plated in 96-well plates and allowed to attach for 24 h, and then treated with various concentrations of DHA or EPA (25, 50,75, 100 μM) for 24 h, 48 h, 72 h and 96 h respectively. After treatment cell growth was assessed and the experiment was performed triplicately.

### Animals and treatment

Animal protocols were approved by Tianjin Medical University Animal Care and Use Committee. The methods were carried out in accordance with the approved guidelines. Forty male Balb/c mice aged at 8 weeks obtained from *Institute of Laboratory Animal Sciences, CAMS & PUMC* (Beijing, China), weighting about 20 g. Mice were maintained in a 12-h light/dark cycle at 22 °C with free access to food and water. After acclimatization for one week, mice were randomly divided into four groups of 10 animals each: control, CCl_4_, fish oil, fish oil/CCl_4_. Control group and CCl_4_ group were fed with AIN 93 diet throughout the period of experiment. The fish oil group and fish oil/CCl_4_ group were fed with AIN 93 diet containing 10% (wt/wt) fish oil (Wuhan Sheng Tian Yu Biotechnology Co., Ltd., Wuhan, China) that contains 33% EPA and 23% DHA. In addition to EPA and DHA, the other components of fish oil include gelatin, glycerin and water. A hepatic fibrosis mice model was established by administration of carbon tetrachloride (CCl_4_, Sigma-Aldrich, St. Louis, MO, USA). Mice in the CCl_4_ group and the fish oil/CCl_4_ group, separately, were administered 5% CCl_4_ (v/v) dissolved in olive oil (0.2 ml/kg body weight) twice per week for 8 weeks via intraperitoneal (ip) injection. Control and fish oil group animals were injected with an equivalent volume of olive oil. After treatment with CCl_4_ for 8 weeks, all of mice were sacrificed under anesthesia with 3% sodium pentobarbital (45 mg/kg, ip). Liver specimens were obtained for analyses of liver functions, mRNA and protein expression of fibrotic indexes by real-time reverse transcription polymerase chain reaction (RT-PCR), Western blot, histology, and immunohistochemistry.

### Measurement of fatty acids

Fatty acids in liver were measured using the method of Kang *et al*.^47^,^48^. Briefly, C17:0 (1 mg/ml in hexane) (Sigma, St. Louis, MO) was added to each sample as an internal standard and the total lipids extracts from liver tissues in each group were based on the methods of Folch^49^. Fatty acid methyl esters were prepared by heating at 90–110 °C for 1 h under BF3/methanol reagent (14% Boron Trifluoride) and analyzed by gas chromatography using a fully automated HP5890 system equipped with a flame ionization detector. The chromatography utilized an Omegawax 250 capillary column (30 m × 0.25 mm I.D.). Peaks were identified by comparison of retention times with external FA methyl ester standard mixtures from NuCheck Prep (Elysian, MN). Results were normalized for weight percent of each FA.

### Hydroxyproline measurement

Commercially available hydroxyproline detection kits were purchased from NanJingJanCheng Biochemical Institute (Nanjing, China). Detection was by colourimetry and hydroxyproline content in liver tissue was determined according to the manufacturer’s instruction.

### Measurement of serum biochemical markers

Blood samples were collected and processed to Serum. Serum levels of alanine aminotransferase(ALT), aspartate aminotransferase(ALP) were measured by commercially available diagnostic kits from Nanjing Jiancheng Institute of Biotechnology (Nanjing, China). The final data are represented as units/liter (U/L).

### Immunofluorescence staining

Cultured cells were fixed with 4% formalin in 4 °C overnight, permeabilized in 1% Triton X-100/PBS, blocked with 5% BSA/TBST for 30 min and incubated with antibodies against YAP/TAZ (Cell Signaling, Danvers, MA, USA), α-SMA (Abcam, Cambridge, United Kingdom) in 4 °C overnight. Samples were washed in PBS before incubation with FITC labeled secondary antibodies (Invitrogen, Grand Island, NY, USA). DAPI (Sigma-Aldrich, St. Louis, MO, USA) was used as a nuclear marker.

### Histological and immunohistochemical analysis

For histopathological evaluation, 10% formalin-fixed, paraffin-embedded sections of the liver were stained with hematoxylin-eosin (HE) and Sirius red. For immunohistochemical analysis, deparaffinized sections were incubated with α-SMA, collagen1 (Abcam, Cambridge, United Kingdom), PCNA and YAP/TAZ (Cell Signaling, Danvers, MA, USA). Briefly, liver tissue sections were deparaffinized, hydrated, heated to 108 °C in citrate buffer for 5 min, depletion of endogenous peroxidase activity, and then blocked with goat serum for 20 min. Next, the slides were treated with primary antibodies α-SMA (1:100), collagen1 (1:500), PCNA (1:800) or YAP/TAZ (1:100), respectively, overnight at 4 °C. An irrelevant isotype rabbit IgG was used as a negative control. The slides were incubated with secondary antibody (HRP-conjugated anti-rabbit IgG), and positive cells were visualized with diaminobenzidine (DAB). The reaction was monitored by microscopy and was terminated when properly developed.

### Isolation of mouse primary HSCs

HSCs were isolated from 32-week old male Balb/c mice or mice treated with control or fish oil diet by *in situ* pronase, collagenase perfusion and Nycodenz gradient as previously reported^50^ . Liver was initially *in situ* digested with 0.05% pronase E (Roche, Mannheim, Germany), 0.03% collagenase type IV (Sigma-Aldrich, St. Louis, MO, USA) and then further digested with collagenase type IV, pronase E and DNase I (Roche, Mannheim, Germany) solution at 37 °C bath shaking for 20 minutes. After hepatocytes were pelleted by centrifugation 50 g for 4 minutes, the supernatant containing nonparenchymal cells was further centrifuged at 500 g for 5 minutes. HSCs were isolated from nonparenchymal cells using 8.2%, 12% and 18% Nycodenz solution (Sigma-Aldrich, St. Louis, MO, USA) at 1450 g and 4 °C without brake for 22 minutes. The purity of HSCs was higher than 95% evaluated by retinoid autofluorescence. Cell viability was determined by the trypan blue exclusion method. HSCs counting were performed with a hemocytometer.

### RNA interference

Human YAP specific and control random small interfering RNAs were chemically synthesized by GenePharma Biological Technology (Shanghai, China). Target sequences of these siRNA are: YAP 1# (S:5′GACGACCAAUAGCUCAGAUTT3′AS: 5′AUCUGAGCUAUUGGUCGUCTT3′), 2# (S:5′GGUGAUACUAUCAACCAAATT3′ AS:5′UUUGGUUGAUAGUAUCACCTT3′), 3# (S:5′CUGCCACCAAGCUAGAUAATT3′ AS: 5′UUAUCUAGCUUGGUGGCAGTT3′), Negative siRNA(S:5′UUCUCCGAACGUGUCACGUTT3′AS:5′ACGUGACACGUUCGGAGAATT3′). Cells were transfected with the siRNAs at 50% confluence using lipofectamine MAX according to the manufacturer’s instructions (Invitrogen, Grand Island, NY, USA). After culturing for 48 h, cells were analyzed by real-time PCR and Western blot to determine knockdown efficiency.

### Western blot analysis

Cells were harvested followed by lysis with 1 × lysis buffer (Cell Signaling, Danvers, MA, USA) with 10 MmNaF and 1 mM PMSF, vortexed and maintained in ice 30 min, then centrifuged at 14,000 rpm, 4 °C for 20 min to obtain supernatants as whole cell lysates. Total protein in the extracts was estimated by the Bradford Assay (Bio-Rad Laboratories, Hercules, CA, USA). Fixed amount of protein was loaded on a polyacrylamide gel followed by transfer to a PVDF membrane (Millipore, Darmstadt, Germany). The membrane was incubated in 5% nonfat dry milk and 0.1% Tween20 in TBS at room temperature for 1 h with shaking, followed by incubation with shaking overnight at 4 °C with antibodies against YAP/TAZ (Cell Signaling, Danvers, MA, USA), YAP (Santa Cruz Biotechnology, Texas, USA), α-SMA, collagen1α1 (Abcam, Cambridge, United Kingdom), and GAPDH diluted in TBS containing 5% milk and 0.1% Tween 20. Signal was detected using the chemiluminescence (ECL) system (Merck Millipore, Darmstadt, Germany).

### Quantitative real-time PCR

Total RNA was isolated with Trizol reagent (Takara, Dalian, China) and the first-strand cDNA was synthesized from total RNA using AMV Reverse Transcriptase (Thermo Fisher Scientific, Basingstoke, UK) according to the manufacturer’s protocol. Real-time PCR was performed in triplicate with SYBR Green master mix (Takara, Dalian, China) for 15 min at 95 °C for initial denaturation, followed by 40 cycles of segments of 95 °C for 30 sec and 60 °C for 30 sec in the LightCycler^®^96 Real-Time PCR System (Roche, Mannheim, Germany). The sequences of primers for real-time PCR are listed in Supplemental Table 4.

### Statistics

Data were expressed as mean ± SEM. All the statistical analysis was performed with the SPSS 13.0 (IBM, Armonk, NY, USA). Statistical analysis was performed using either Student’s t-test (two-group comparison) or one-way analysis of variance (more than two groups) followed by *post hoc* comparison, and differences with *p* < 0.05 were considered significant.

## Additional Information

**How to cite this article**: Zhang, K. *et al*. ω-3 PUFAs ameliorate liver fibrosis and inhibit hepatic stellate cells proliferation and activation by promoting YAP/TAZ degradation. *Sci. Rep.* **6**, 30029; doi: 10.1038/srep30029 (2016).

## Supplementary Material

Supplementary Information
