# Supplementary material for: ω-3 PUFAs ameliorate liver fibrosis and inhibit hepatic stellate cells proliferation and activation by promoting YAP/TAZ degradation
Source: Sci Rep. 2016 Jul 20;6:30029. doi: 10.1038/srep30029 (PMC4951777; doi:10.1038/srep30029)
Supplement: Supplementary Information [file srep30029-s1.pdf]

ω-3 PUFAs ameliorate liver fibrosis and inhibit hepatic stellate cells proliferation and activation by promoting YAP/TAZ degradation

Kun Zhang[1,*], Yanan Chang[1,*], Zhemin Shi[1,*], Xiaohui Han[1], Yawei Han[1], Qingbin Yao[1], Zhimei Hu[1], Hongmei Cui[1], Lina Zheng[1], Tao Han[2,3,4] & Wei Hong[1]

[1]Department of Histology and Embryology, School of Basic Medical Sciences, Tianjin Medical University, Tianjin 300070, China

[2]The Third Central Clinical College of Tianjin Medical University, Tianjin 300170, China

[3]Department of Hepatology, Tianjin Third Central Hospital, Tianjin 300170, China

[4]Tianjin Key Laboratory of Artificial Cells, Tianjin 300170, China

[*]These authors contributed equally to this work.

Correspondence and requests for materials should be addressed to T.H. (email: jichu022@tmu.edu.cn) or W.H. (email: hongwei@tmu.edu.cn)

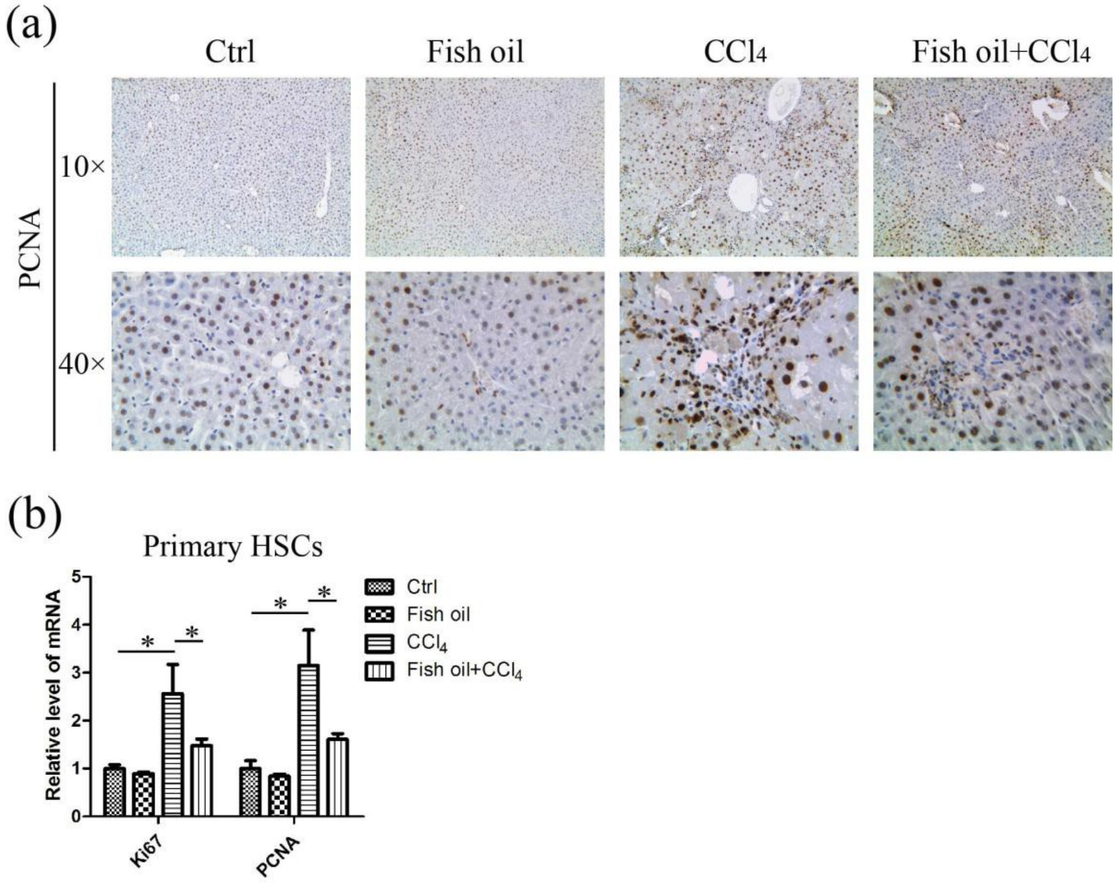

Supplemental Fig. 1. ω-3 PUFAs inhibit the proliferation of activated hepatic stellate cells *in vivo*. (a) Liver sections from mice of the indicated group were immunostained with anti-PCNA antibody. (b) Total RNA from HSCs immediately isolated from liver tissues in control, fish oil, CCl_4 and fish oil/CCl_4 mice was extracted and subsequently used for the detection of the mRNA of *Ki67* and *PCNA* by real-time PCR analysis. Data presented are mean ± SEM for triplicate experiments.**P*<0.05.

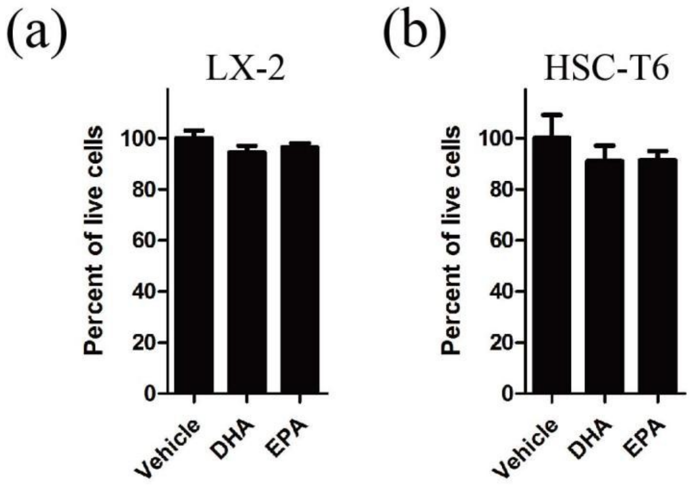

Supplemental Fig. 2. DHA and EPA exhibit no cytotoxic effect on LX-2 and HSC-T6 cells. (a, b) LX-2 (a) and HSC-T6 (b) cells growing in medium were treated with 100 μM either DHA or EPA, respectively, for 6 h and subsequently trypan blue staining was performed. The cell numbers were counted to measure the viability. Treatment with ethanol was used as a vehicle control. Data presented are mean ± SEM for triplicate experiments.

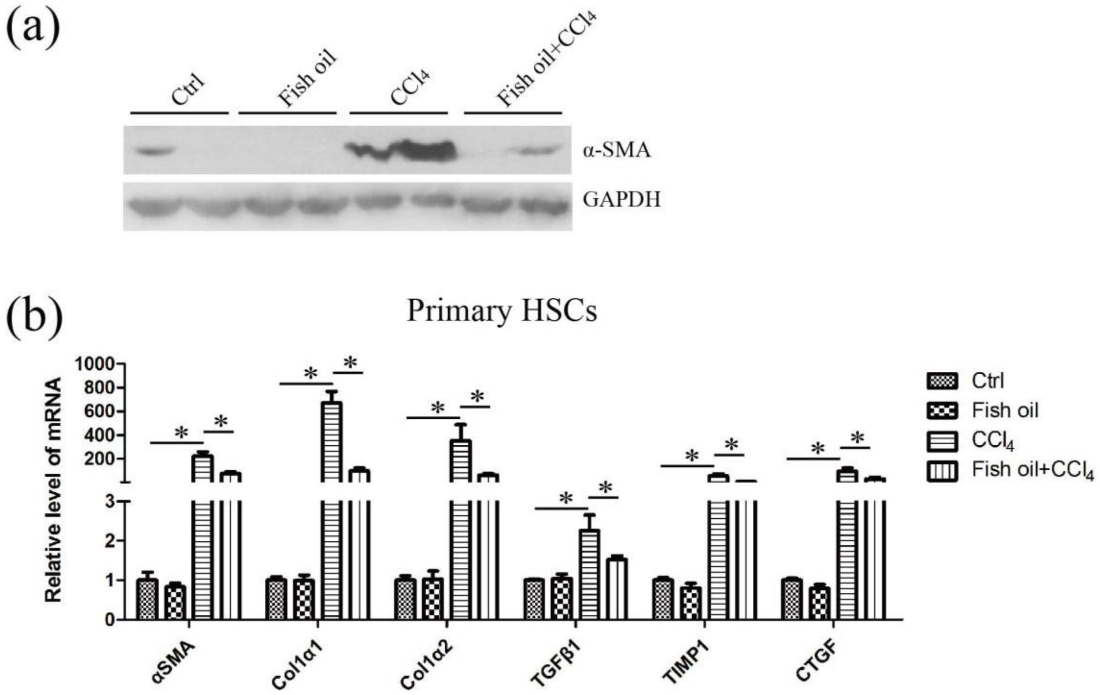

Supplemental Fig. 3. Fish oil down-regulates the expression of fibrogenic genes *in vivo*. (a) Total protein of HSCs immediately isolated from liver tissues in control, fish oil, CCl_4 and fish oil/CCl_4 mice was extracted and used for detecting α-SMA by western blot. GAPDH served as the loading control. The gels were cropped and the full-length gels are presented in Supplementary Fig. 5. (b) Total RNA of HSCs immediately isolated from liver tissues in control, fish oil, CCl_4 and fish oil/CCl_4 mice was extracted and used for detection of the fibrogenic genes, including *α-SMA, col1α1, col1α2, tgf-β1, timp1*and *ctgf* by real-time PCR. The data are expressed as the mean ± SEM for triplicate experiments. *P<0.05.

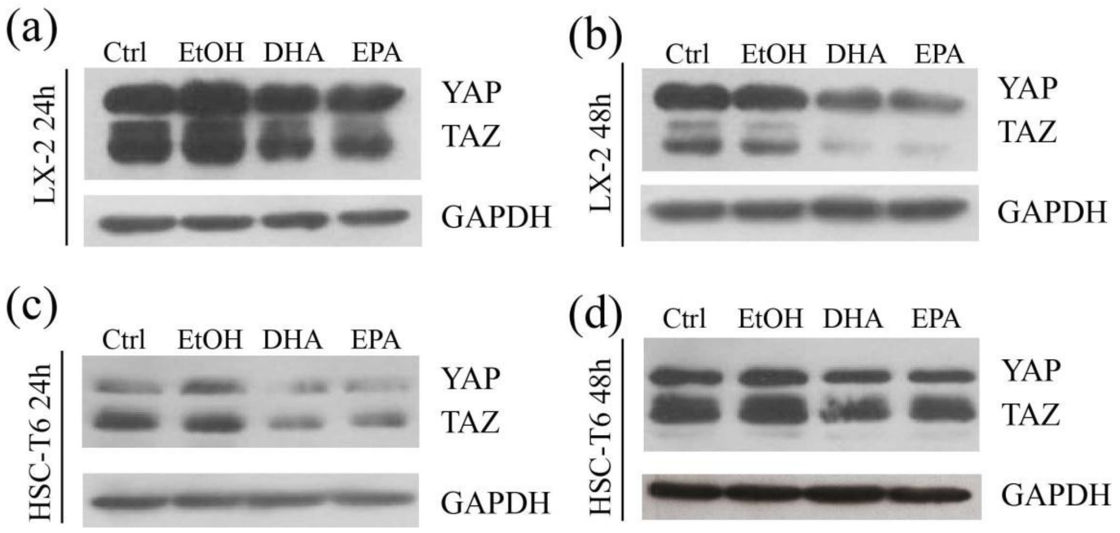

Supplemental Fig. 4. DHA and EPA decrease the protein level of YAP/TAZ in LX-2 and HSC-T6 cell lines. (a-d) LX-2 (a, b) and HSC-T6 (c, d) cells were exposed for 24 or 48 h to media containing 10% fetal bovine serum, ethanol, 75 μM DHA or EPA. Cell lysates from both cell lines were subsequently assessed by immunoblot to determine the protein levels of YAP/TAZ. GAPDH was used as internal control. The gels were cropped and the full-length gels are presented in Supplementary Fig. 6. The data are expressed as the mean ± SEM for triplicate experiments. *$P<0.05$.

Fig. 1b

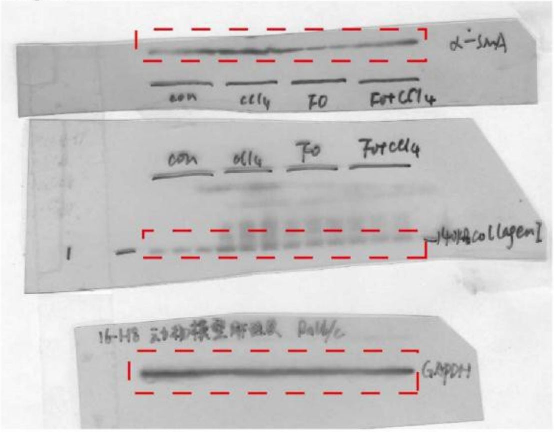

Fig. 3e-h

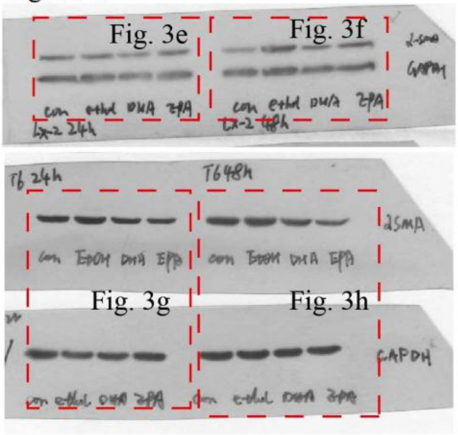

Fig. S2a

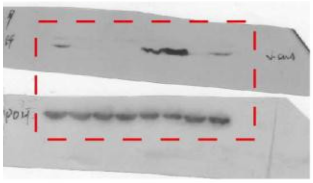

Fig. 5a

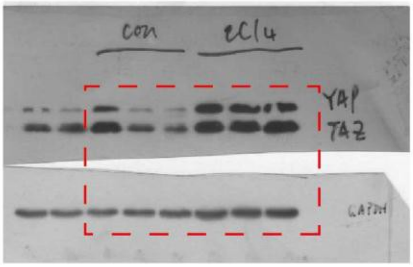

Fig. 5f

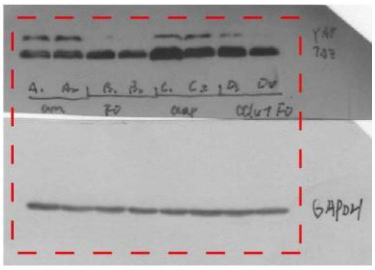

Supplemental Fig. 5. Uncropped, unprocessed images of blots and gels.

Fig. 6a,b and Fig. S3a,b

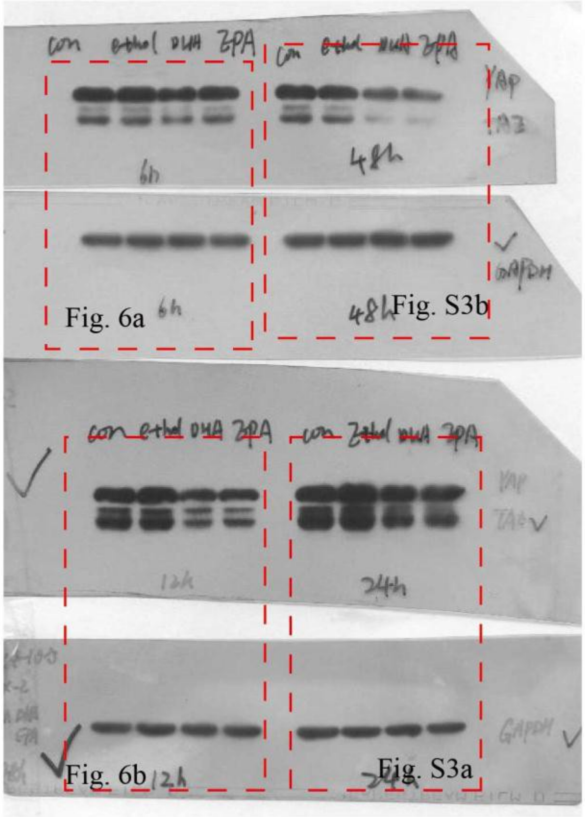

Fig. 6c and Fig. S3c

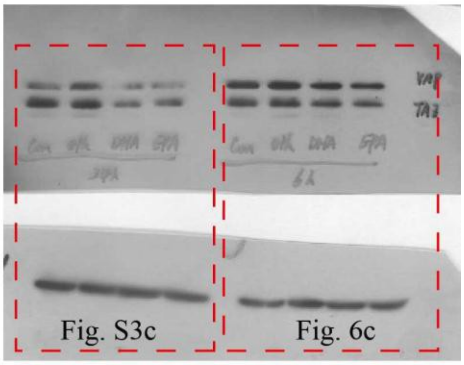

Fig. 6d

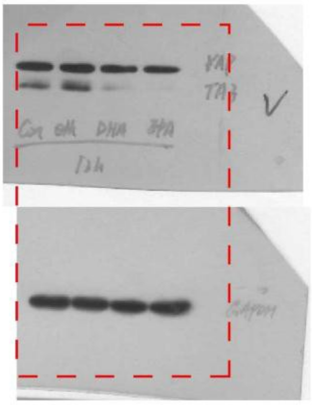

Fig. S3d

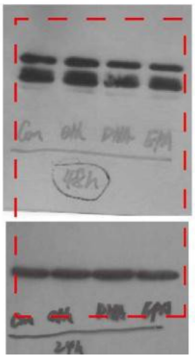

Fig. 7b

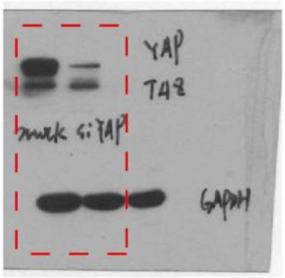

Supplemental Fig. 6. Uncropped, unprocessed images of blots and gels.

Fig. 8a

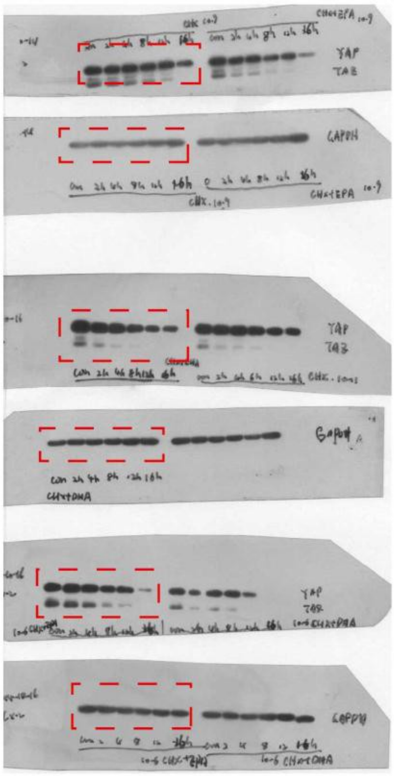

Fig. 8b

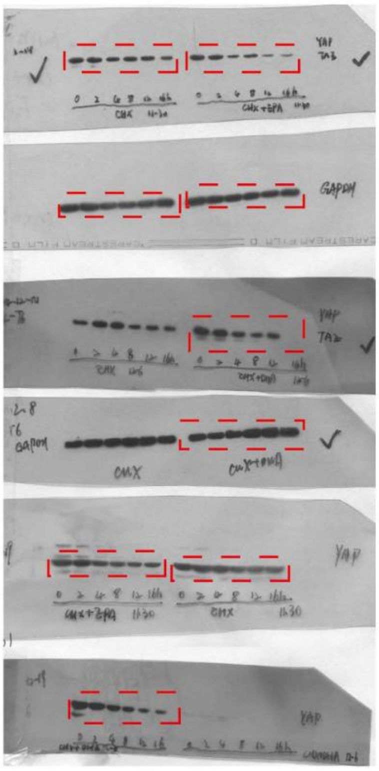

Fig. 8c

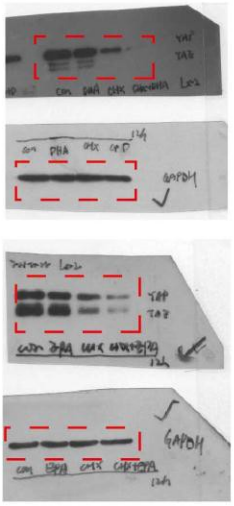

Fig. 8e

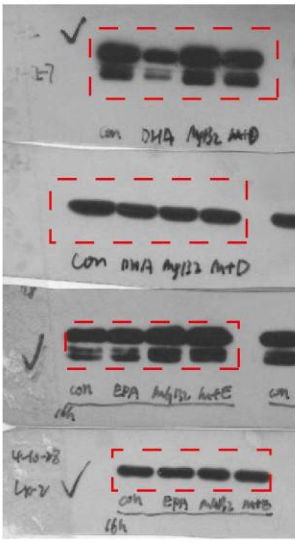

Fig. 8d

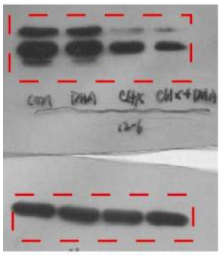

Fig. 8d and 8f

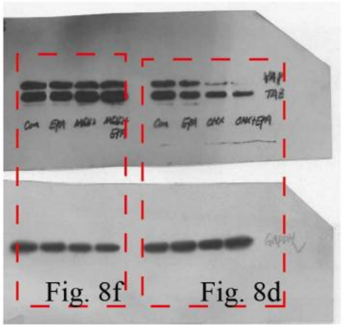

Fig. 8f

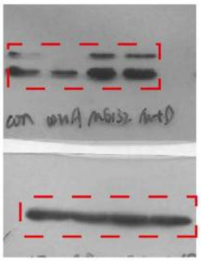

Supplemental Fig. 7. Uncropped, unprocessed images of blots and gels.

Supplemental Tab.1. Mouse liver tissue fatty acid composition in the four groups (weight % of total fatty acids)

PUFA	Control	Fish oil	CCl_4	Fish oil + CCl_4
LNA (18:2 n-6)	9.34±0.98	3.75±1.64*	8.92±0.92	4.13±1.38##
AA (20:4 n-6)	7.28±1.22	4.36±0.68*	6.49±0.96	3.28±0.56#
DTA (22:4 n-6)	0.19±0.05	0.09±0.02*	0.20±0.02	0.04±0.03#
EPA (20:5 n-3)	0.33±0.08	1.09±0.18**	0.49±0.14	0.93±0.12#
DPA (22:5 n-3)	0.24±0.05	0.98±0.12**	0.23±0.06	1.12±0.15##
DHA (22:6 n-3)	3.28±1.13	12.35±1.48**	4.32±0.87	13.65±2.17##

Values are means ± SEM, n=5 mice per group. $*/^{\#}p<0.05$, $**/^{\#\#}p<0.01$. $*p<0.05$ compared with control group. $^{\#}p<0.05$ compared with CCl_4 group. We used analysis of variance (ANOVA) test among groups and Student's *t* test between two groups. All statistical analyses were performed using SPSS version 13.0 software and $p<0.05$ indicated statistical significance.

Supplemental Tab.2. Serum levels of ALT, AST

Group	ALT (U/L)	AST (U/L)
Control group	28.3±7.1	37.4±8.2
Fish oil group	27.5±10.1	33.7±11.4
CCl4 group	258.3±47.2*	281.6±65.3*
CCl4+fish oil group	128.3±31.5#	103±43.8#

Values are means ± SEM, n=5 mice per group. *p<0.05 compared with control group. #p<0.05 compared with CCl_4 group. We used analysis of variance (ANOVA) test among groups and Student's *t* test between two groups. All statistical analyses were performed using SPSS version 13.0 software and p<0.05 indicated statistical significance.

Supplemental Tab.3. The number of hepatic stellate cells in the liver of one Balb/c mouse

Number	Control	Fish oil	CCl_4	Fish oil + CCl_4
HSCs	$(67 \pm 12) \times 10^5$	$(62 \pm 18) \times 10^5$	$(203 \pm 42) \times 10^5$*	$(136 \pm 27) \times 10^{5\#}$

Values are means ± SEM, n=3 mice per group. *$p<0.05$ compared with control group. $^{\#}p<0.05$ compared with CCl_4 group. We used analysis of variance (ANOVA) test among groups and Student's *t* test between two groups. All statistical analyses were performed using SPSS version 13.0 software and $p<0.05$ indicated statistical significance.

Supplemental Tab.4. The sequences of primers for real-time PCR

Gene symbol	Forward 5' - 3'	Reverse 5 '- 3'
β-actin (mouse)	ATGCCACAGGATTCCATACCCAAGA	CTCTAGACTTCGAGCAGGAGATGG
Col1α1 (mouse)	ATCGGTCATGCTCTCTCCAAACCA	ACTGCAACATGGAGACAGGTCAGA
Col1α2 (mouse)	CCTTTGTCAGAATACTGAGCAGC	GTAACTTCGTGCCTAGCAACA
Col3α1 (mouse)	TGCTCCAGTTAGCCCTGCAA	GGTCCTGCAGGCAACAGTGGTTC
Col4α5 (mouse)	CTCCCTTACCGCCCTTTTCTC	AGGCGAAATGGGTATGATGGG
CTGF (mouse)	ATCCAGGCAAGTGCATTGGTA	GGGCCTCTTCTGCGATTTC
α-SMA (mouse)	TCGGATACTTCAGCGTCAGGA	GTCCCAGACATCAGGGAGTAA
TIMP-1 (mouse)	TCCGTCCACAAACAGTGAGTGTCA	GGTGTGCACAGTGTTTCCCTGTTT
Mmp2 (mouse)	GTGTTCTTCGCAGGGAATGAG	GATGCTTCCAAACTTCACGCT
TGF-β1 (mouse)	TGTGTTGGTTGTAGAGGGCAAGGA	TTTGGAGCCTGGACACACAGTACA
YAP (mouse)	CCACTGTTAAGAAAGGGATCGG	CCCTGATGATGTACCACTGCC
TAZ (mouse)	TTACAGCCAGGTTAGAAAGGGCTC	GAAAATCACCACATGGCAAGACCC
Ki67 (mouse)	CATCCATCAGCCGGAGTCA	TGTTTCGCAACTTTCGTTTGTG
PCNA (mouse)	TTTGAGGCACGCCTGATCC	GGAGACGTGAGACGAGTCCAT
β-actin (rat)	CGCTCGGTCAGGATCTTC	CGGCATTGTCACCAACTG
Col1α1 (rat)	ATCGGTCATGCTCTCTCCAAACCA	ACTGCAACATGGAGACAGGTCAGA
Col1α2 (rat)	CCTTTGTCAGAATACTGAGCAGC	GTAACTTCGTGCCTAGCAACA
Col3α1 (rat)	TGCTCCAGTTAGCCCTGCAA	GGTCCTGCAGGCAACAGTGGTTC
Col4α5 (rat)	CTCCCTTACCGCCCTTTTCTC	AGGCGAAATGGGTATGATGGG
CTGF (rat)	CCGCCAACCGCAAGATT	TCGGGAAGGGGCAGTCA
α-SMA (rat)	GATCACCTGCCCATCAGG	TGTGCTGGACTCTGGAGATG
TIMP-1 (rat)	TCTGATCTGTCCACAAGCAAT	TCCTGGTTCCCTGGCATAATC
TGF-β1 (rat)	AGGTGTTGAGCCCTTTCCAG	CAAAGACATCACACACAGTA
YAP (rat)	TCGGTACTGGCCTGTCGCGA	CGTGCCCATGAGGCTTCGCA
TAZ (rat)	GAGGAAGGGCTCGCTTTTGT	ATGTTGACCTCGGGACTTTGG
GAPDH (human)	GGCATGGACTGTGGTCATGAG	TGCACCACCAACTGCTTAGC
Col1α1 (human)	TCCACATGCTTTATTCCAGCAATC	CCCGGGTTTCAGAGACAACTTC
Col3α1 (human)	AGCCTTGCGTGTTCGATAT	GAAGATGTCCTTGATGTGC
Col4α5 (human)	TTCAGCGTTTCTGACTGAGG	AGAGCATCCAGCCATTCATT
CTGF (human)	TGCTTTGAACGATCAGACAA	CTTGTGGCAAGTGAATTTCC
α-SMA (human)	GCCATGTTCTATCGGGTACTTC	CAGGGCTGTTTTCCCATCCAT
Mmp2 (human)	GTGTTCTTCGCAGGGAATGAG	GATGCTTCCAAACTTCACGCT
TGF-β1 (human)	TGTTGGACAGCTGCTCCACCT	GGCAGTGGTTGAGCCGTGGA
YAP (human)	CCAGGAATGGCTTCAAGGTA	CTCGAACCCCAGATGACTTC
TAZ (human)	GTGATTACAGCCAGGTTAGAAAG	CCATCACTAATAATAGCTCAGATC
